# Two choices good, four choices better: For measuring stereoacuity in children, a four-alternative forced-choice paradigm is more efficient than two

**DOI:** 10.1371/journal.pone.0201366

**Published:** 2018-07-30

**Authors:** Kathleen Vancleef, Jenny C. A. Read, William Herbert, Nicola Goodship, Maeve Woodhouse, Ignacio Serrano-Pedraza

**Affiliations:** 1 Institute of Neuroscience, Newcastle University, Newcastle-upon-Tyne, United Kingdom; 2 Faculty of Psychology, Complutense University of Madrid, Madrid, Spain; University of Montreal, CANADA

## Abstract

**Purpose:**

Measuring accurate thresholds in children can be challenging. A typical psychophysical experiment is usually too long to keep children engaged. However, a reduction in the number of trials decreases the precision of the threshold estimate. We evaluated the efficiency of forced-choice paradigms with 2 or 4 alternatives (2-AFC, 4-AFC) in a disparity detection experiment. 4-AFC paradigms are statistically more efficient, but also more cognitively demanding, which might offset their theoretical advantage in young children.

**Methods:**

We ran simulations evaluating bias and precision of threshold estimates of 2-AFC and 4-AFC paradigms. In addition, we measured disparity thresholds in 43 children (aged 6 to 17 years) with a 4-AFC paradigm and in 49 children (aged 4 to 17 years) with a 2-AFC paradigm, both using an adaptive weighted one-up one-down staircase.

**Results:**

Simulations indicated a similar bias and precision for a 2-AFC paradigm with double the number of trials as a 4-AFC paradigm. On average, estimated threshold of the simulated data was equal to the model threshold, indicating no bias. The precision was improved with an increasing number of trials. Likewise, our data showed a similar bias and precision for a 2-AFC paradigm with 60 trials as for a 4-AFC paradigm with 30 trials. Trials in the 4-AFC paradigm took slightly longer as participants scanned more alternatives. However, the 4-AFC task still ended up faster for a given precision.

**Conclusion:**

Bias and precision were similar in a 4-AFC task compared to a 2-AFC task with double the number of trials. However, a 4-AFC paradigm was more time efficient and is therefore recommended.

## Introduction

Possibly the most fundamental way of characterising a perceptual system is to measure the detection threshold: the minimum signal strength which can be detected. In principle, this can be measured by asking the subject to report whether or not a signal was present in a given stimulus, and defining a threshold as the point where the signal is detected on a specified fraction of trials. However, performance on such yes/no tasks is influenced not only by the detection threshold, but also by the subject’s decision criterion for reporting a signal. In forced-choice tasks a subject is instead asked to report which of several alternative stimuli contained the signal. This produces an estimate of detection threshold unbiased by a decision criterion [1,2]. Accordingly, forced-choice tasks are among the most widely-used psychophysical procedures.

Despite being unbiased by a decision criterion, forced-choice tasks still suffer from other problems. For example, thresholds can be contaminated by response bias [3] (temporal or position bias), although the effect of this bias on threshold estimation is negligible [4]. Notably, a high number of trials is necessary to obtain reliable threshold estimates, resulting in long experiment durations (up to several hours if several thresholds are required) and requiring strong motivation and commitment from the subject. This makes it difficult to obtain reliable thresholds in young children. However, a reduction in the number of trials decreases the precision of the threshold estimate [1,4,5].

An alternative way to increase precision is to increase the number of alternatives in forced-choice tasks. Although a forced-choice task with 2 alternatives (2-AFC) is probably the most commonly used in the vision research community [1], increasing the number of alternatives reduces the guessing rate and therefore makes every trial more informative. A smaller guessing rate also increases the slope of the psychometric function and improves efficiency of the threshold estimates [6]. These theoretical advantages increase monotonically with the number of alternatives *m*. However, in reality, there must be a point at which *m* becomes too large to be practical.

A few studies have looked into directly comparing *m*-AFC tasks. Shelton and Scarrow compared two and three alternatives in an auditory detection task [7]. Although similar threshold estimates were obtained, stability of the threshold estimates was better in the 3-AFC compared to the 2-AFC task. The 3-AFC task also minimized between-subject variability. Leek, Hanna, and Marshall compared two, three, and four interval forced-choice tasks in a simulation study [8]. The authors concluded four alternatives gave the most accurate results with the least variability. The procedure was also the most efficient. Jäkel and Wichmann also observed that for contrast sensitivity, a 4-AFC task was 3.5 times more efficient (time needed to get smaller confidence intervals) than 2-IFC task, and is the recommended task for naïve observers. Also, in food flavour discrimination, a 4-AFC task was found to be more powerful than a 2-AFC or 3-AFC task [9]. A quick contrast sensitivity function method also seems to benefit from increasing the number of alternatives: in simulations and in a behavioural experiment Hou et al. showed that a 10-AFC task has the highest efficiency compared to 2, 4, 8 and 16 alternatives [6].

In the previous studies, the subjects were all adults and even the naive observers completed a large number of trials over the course of the experiment (20,000 in [9]; 300 in [7]). Therefore conclusions cannot be generalized to a child population with whom fewer than 100 trials can be obtained. Furthermore, young children (under the age of 11 years or so) have a shorter attention span than adults, especially on tasks with a high cognitive load [10]. Trials with more alternatives are inherently more complex and may require more time to inspect the possible alternatives, resulting in longer experiment duration than the same number of 2-AFC trials. In children, with limited cognitive and attentional resources, the increased complexity of 4- or higher *m*-AFC trials could outweigh the other known advantages.

To address this question, in the current study, we use simulations and data from psychophysical experiments with children. We examine whether previous conclusions in adults, that 4-AFC tasks yield more precise threshold estimates than 2-AFC, also hold in young children. In addition, we will investigate time-efficiency of both paradigms, by comparing the total duration of a 2-AFC experiment and a 4-AFC experiment that achieve the same levels of precision of threshold estimates.

## Materials and methods

The task used in our experiments, and modelled in our simulations, was a disparity detection task suitable for assessing stereoacuity. Subjects are asked to find a “target” stimulus amongst either 1 or 3 “distractor” stimuli (2-AFC or 4-AFC), presented simultaneously in different spatial locations. The distractor stimuli have uniform binocular disparity, depicting a flat surface, whereas the target stimulus has a disparate square patch which appears to be sticking out in depth.

### Simulations

**Fig 1:**
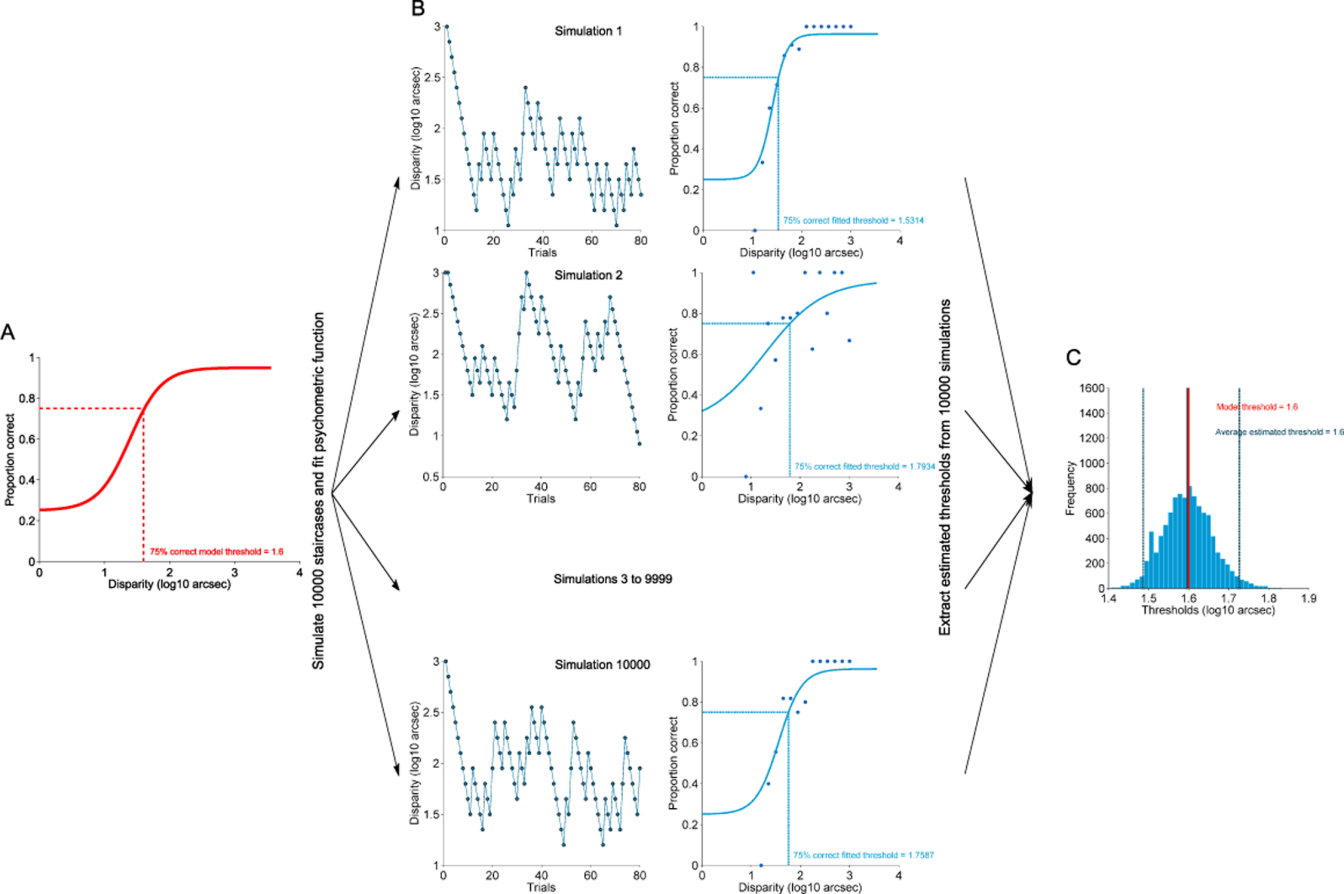
Illustration of simulation procedure for a 4-AFC paradigm. A) Model function used in this example (model threshold *θ* = 1.6 log_10_ arcsec). Each staircase (B) follows the rules described in the main text: starting at 3 log_10_ arcsec, decreasing the disparity by 0.15 log_10_ arcsec after a correct answer and increasing disparity by 0.45 log_10_ arcsec after an incorrect answer. Each row in (B) shows an example of one simulation with 80 trials. The left plots in (B) show the staircases: the ‘presented’ disparities for each simulation of the 80 trials. The right plots show the probability correct as a function of disparity, with the dots being the simulated data and the line the fitted psychometric function to the simulated data. From this fitted psychometric function the threshold value was estimated. After 10000 repeats, the estimated thresholds can be presented in an histogram (C) to show the distribution of likely thresholds that can result from the 4-AFC staircase procedure with this model psychometric function (A). The average of this ditribution can be compared with the model threshold as an indication of bias and precision of the 4-AFC staircase procedure.

We simulated a staircase procedure for a 2-AFC and a 4-AFC task with a range of model thresholds, and for a variety of trial numbers. Model thresholds (θ) ranged from 10 to 500 arcsec, i.e. 1 to 2.7 log_10_ arcsec in steps of 0.1 log_10_ arcsec, covering most of the range of stereoacuity in normal observers including children [11]. Trial numbers ranged from 10 to 80 trials in steps of 5 trials. For each combination, 10000 simulated experiments were run following the staircase procedure described below (Fig 1), which was the same procedure as in the behavioural experiments. The first trial started at a disparity of 3 log_10_ arcsec, then the disparity decreased by 0.15 log_10_ arcsec after a correct answer and increased by 0.45 log_10_ arcsec after an incorrect answer. In each simulated experiment the psychometric function used as a model was a logistic function adapted from [12]:

$$\Psi \left(x\right)=\gamma +\frac{1-\lambda -\gamma }{1+exp\left[\beta \left(\alpha -x\right)\right]}$$ (1)

with x is the disparity in log_10_ arcsec. We used a model guessing rate (γ) of 0.25 for 4-AFC and 0.5 for 2-AFC task. The model lapse rate (λ) was fixed at λ = 0.05*(1-γ) [1], where 0.05 is the probability of making a lapse, so λ = 0.025 for 2-AFC and λ = 0.0375 for 4-AFC. In previous work, we have found the average probability of children making a lapse to be in the range of 1% to 2.25% for both 2-AFC and 4-AFC [13], and simulations show that lapse rates within +-1% of the true value do not bias results [14]. The location and slope parameters α and β are defined as follows:

$$\alpha =\theta +\frac{1}{\beta }ln\left[\frac{1-\lambda -\pi }{\pi -\gamma }\right]$$ (2)

$$\beta =\frac{2}{\sigma }ln\left[\frac{1-\lambda -\gamma -\delta }{\delta }\right]$$ (3)

where *π* is the probability of correct responses associated with the threshold *θ* (in log units). We used *π* = 0.75 for both, 2AFC and 4AFC tasks; and *σ* is the spread value defined as:

$$\sigma =\Psi^{-1}\left(1-\lambda -\delta \right)-\Psi^{-1}\left(\gamma +\delta \right)$$ (4)

where *δ* is used to calculate *σ* with a desired central range of the psychometric function:

$$\delta =\frac{100-CR}{200}*\left(1-\lambda -\gamma \right)$$ (5)

where *CR* is the width of the central range of the psychometric function. Thus, for *CR* = 95% (we used this range in all fittings and simulations), we can express the relationship between beta and sigma as follows:

β=7.327/σ (6)

Based on previous research showing similar values for adults and children, the spread value *σ* used for the model function (for both 2AFC and 4AFC) was set at 1.8 log_10_ arcsec. This is slightly higher than average, so simulates a participant with poor discrimination [13]. Finally, for each simulated experiment we fitted to the data the same Eq 1 used as a model with two free parameters, the threshold *θ* and the spread value *σ* (see Fig 1B). The fitted threshold *θ* was constrained to lie between 1 and 1000 arcsec (0 and 3 log_10_ arcsec) and the fitted *σ* between 0 and 5. The lapse rate and guessing rate of each fit were fixed at the same values as the model function. The average and standard deviation of the thresholds fitted to 10000 sets of simulated data were calculated for each combination of model threshold, number of trials and task (see Fig 1C). Subsequently, we calculated bias by subtracting the model threshold from the average of the fitted thresholds. The standard deviation of the fitted thresholds gives a lower bound on the precision of such procedures in humans.

### Experiments

#### Design

Subjects performed a disparity detection task in which they indicated which stimulus showed a square that was standing out in depth. In a between-subjects design, subjects were either given 2 alternatives (2-AFC) or 4 alternatives (4-AFC) to choose from.

#### Subjects

**Fig 2:**
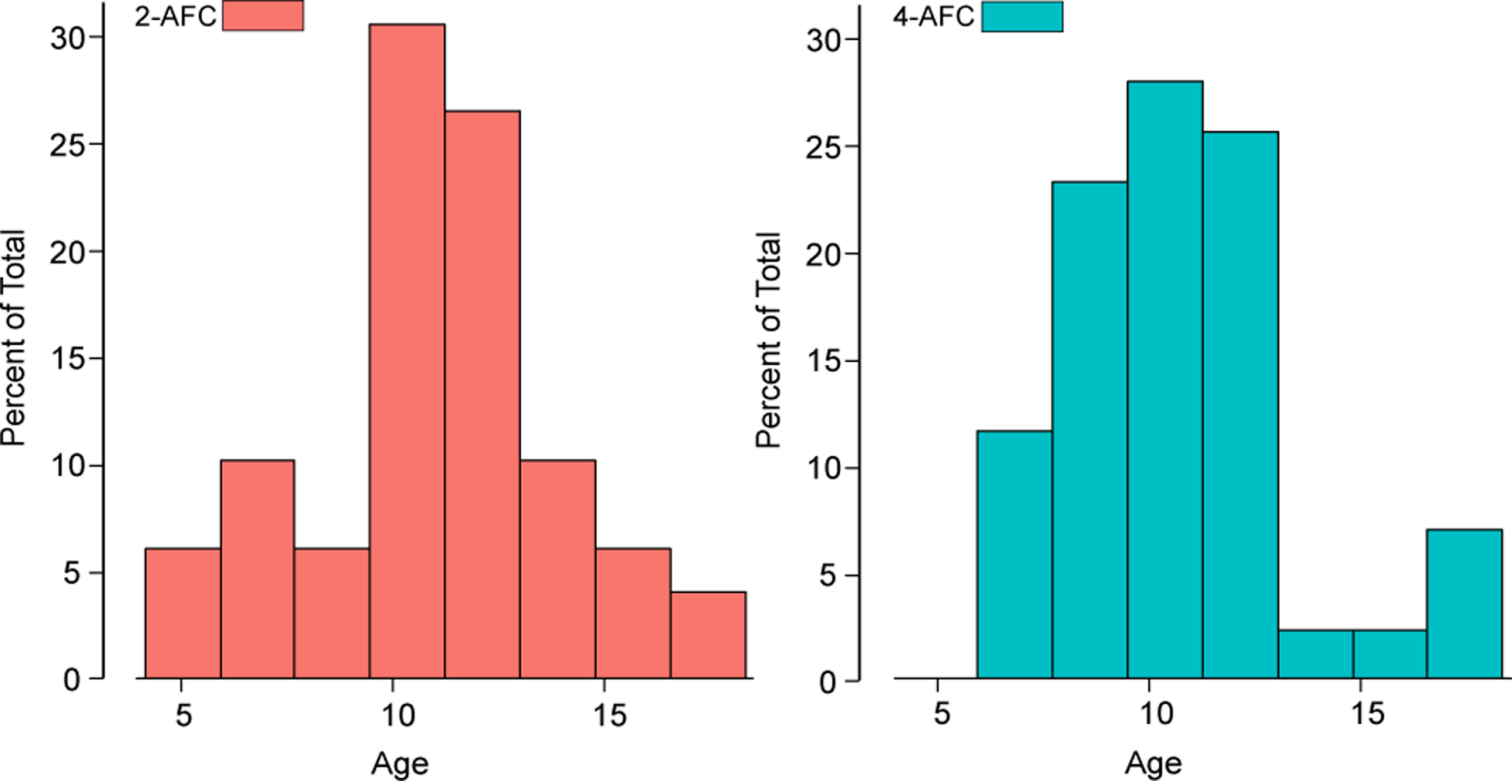
Age distributions of participants in both experiments. The age distribution for the2-AFC task are presented on the left and for the 4-AFC on the right.

A total of 92 children participated in the experiments. 49 children completed the 2-AFC task and 43 different children completed the 4-AFC task. All subjects completed 80 trials. Ages were similarly distributed in both experiments with an average age of 11 years in the 2-AFC task and 10.58 years in the 4-AFC task (see Fig 2, Kolmogorov-Smirnov test, D = 0.21, p = .28). Because we aimed to study measurement of stereovision in the general population, no children were excluded based on eye pathology, but children were asked to wear their habitual correction. Parents or guardians provided written consent for the child. The study protocol was compliant with the Declaration of Helsinki and was approved by the Ethics Committee of the Newcastle University Faculty of Medical Sciences (approval number 00625).

#### Apparatus and set-up

Stimuli were presented on a 23-inch passive 3D monitor (D2367PH, AOC) with a refresh rate of 60 Hz and a spatial resolution of 1920 x 1080 pixels (52 x 29 cm). The 3D stimuli were presented using the line-interleaved stereo mode of Psychotoolbox’s Psychimaging function [15–17]. Left and right images are separated by circular polarized 3D glasses (distributed by Sky 3D, www.sky.com). Children were seated at 90 cm from the monitor (so a pixel subtended 60.4 arcsec on average) with their head in a forehead and chin rest (UHCOTech HeadSpot, Houston, USA, https://www.opt.uh.edu/research/uhcotech/headspot/). In the 2-AFC task, subjects indicated their response by pressing the left or right button of a standard computer mouse. In the 4-AFC task, subjects responded via a ResponsePixx Handheld (VPixx Technologies Inc. Montreal, Canada, https://vpixx.com/). This has five buttons positioned in a quincunx (the number five on a dice). The four corner buttons corresponded with the four spatial locations of the stimuli (where the target could appear); the centre button was not used in the experiment. Data were collected on a DELL workstation (Intel(R) Core(TM) i3 CPU 540 @3.07GHz, 4GB RAM, 64- bit Operating System, Windows 7), with a GeForce GTX 460 graphics card (NVIDIA), running MATLAB R2012a, 64-bit (Mathworks, https://uk.mathworks.com/) and Psychophysics Toolbox extensions [15–17] in a dimmed area at a local science museum (Centre for Life: http://www.life.org.uk/).

#### Stimuli

**Fig 3:**
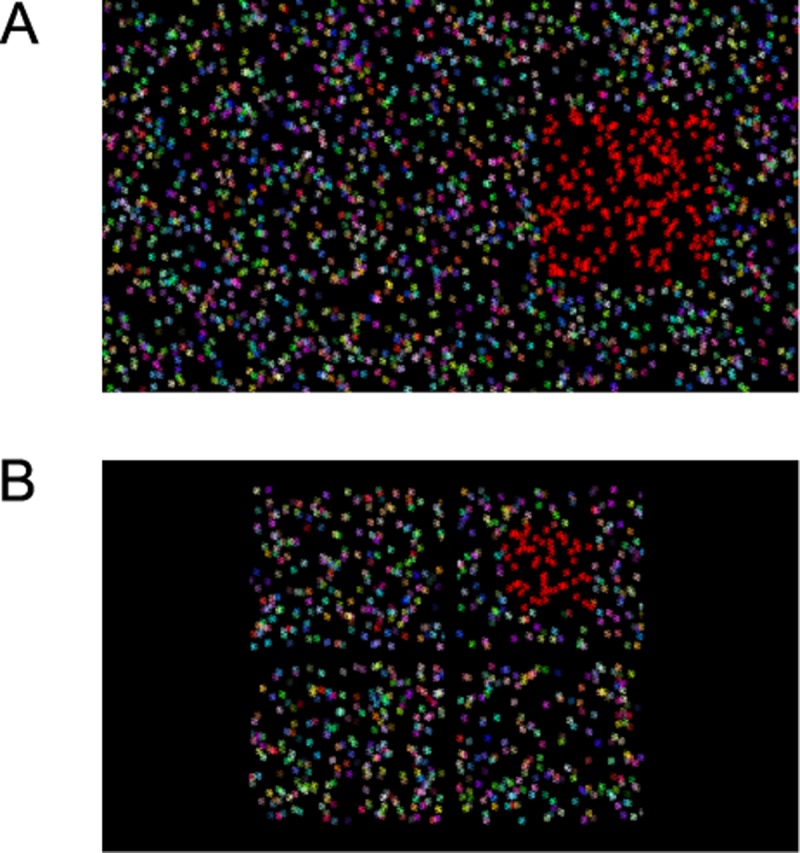
Full screen examples of the stimuli in the 2-AFC and the 4-AFC task. Panel (A) shows a practice trial of the 2-AFC task with line-interleaved elliptic dots. Right side shows the target stimulus: a squared patch standing out in depth from the background. Left side shows the distractor stimulus with only one depth plane. In addition to the disparity difference the target area is also presented in red. This colour/luminance cue is removed after the practice trials. (B) Example of the 4-AFC task with the target stimulus in the right upper quadrant. See dimensions in the main text.

We presented dynamic random-dot stereograms that consisted of brightly-coloured dots on a black background (Fig 3). Dot colour was generated by selecting the R, G and B values independently from a uniform distribution between minimum and maximum luminance with Psychtoolbox’s “Screen(‘DrawDots’)” function. The dots were generated as circles of 10 pixels in diameter with high-quality anti-aliasing to allow sub-pixel disparities. On the screen the dots appeared as ellipses because of the line interleaving. Dots had a width of 10 pixels and a height of 20 physical pixels (10.6 × 20.12 arcmin). The position and colour of dots was random and updated every frame at 60 Hz.

In the 2-AFC task, the target was a random dot stereogram of 8.4 **×** 8.4 deg with crossed disparities presented on top of a surround composed of random dots with uncrossed disparities. The stimulus was presented on the left or right side of the centre of the screen at 9.3 degrees eccentricity (see Fig 3A). In the 4-AFC task, the target was a random dot stereogram of 4.3 **×** 4.3 deg with crossed disparities presented in one of the four corners of the screen at an eccentricity of 6.5 deg (see Fig 3B). The target was located in the centre of the background, a rectangle of random dots with uncrossed disparities (9.3 **×** 7.4 deg, W **×** H). Thus, target and background had equal and opposite disparity relative to the screen. This procedure reduces monocular cues that could be present for high disparities [18]. The stimulus disparity was defined as the relative disparity between the target and background. Presentation time was unlimited and each stimulus was displayed until the child made a response.

#### Staircase procedure

We manipulated disparity following an adaptive weighted one-up one-down staircase (see a simulation study about adaptive weighted one-up one-down staircases in [5]). The staircase started with a practice trial at a disparity of 3 log_10_ arcsec (or 1000 arcsec). In the first trial, in addition to the disparity, all target dots were presented in red at maximum luminance (see Fig 3). This non-stereo colour/luminance cue was added to the practice trial to ease understanding of the task. In the subsequent trials no additional colour/luminance cue was presented and the stimuli could only be discriminated based on disparity. Following each correct answer disparity was decreased by 0.15 log_10_ arcsec. Following each incorrect answer disparity was increased with three times this value or 0.45 log_10_ arcsec. This procedure resulted in a targeted probability correct of 0.75. No feedback was provided during the experiment.

#### Fitting and calculating dependent variables

First we calculated the proportion of correct responses for each value of the disparities presented to the subject. Then, we fitted psychometric functions to subsets of trials: the first 15, 20, 25 … 75, 80 trials of each subject. The combination of 92 subjects and 14 levels of number of trials resulted in 1288 fits. For each of these fits we estimated the disparity threshold (the disparity that corresponds to the probability of correct responses of *π* = 0.75 for both, 2AFC and 4AFC tasks), threshold uncertainty, bias, precision, average trial duration, and total experiment duration.

We estimated the threshold values by fitting to the data the logistic function specified in Eq (1) [11]. The maximum likelihood criterion was used to determine the best fitting psychometric function with two free parameters *θ* and *σ*. Pilot experiments informed us about appropriate starting values for these parameters that were set 1.59 log_10_ arc for *θ* and 1.8 log_10_ arcsec for *σ* [13]. Fitted parameters were constrained to stay within the 0 to 3 log_10_ arcsec limits for *θ* and 0 to 5 log_10_ arcsec for *σ*.

The standard deviation of threshold estimates for each subject, was determined by simulating the same experiment but using the psychometric function fitted to the subject’s data (of 15–80 trials) as the model function. An experiment was simulated in which probabilities for correct or incorrect answer were derived from that model function. Stimulus levels were chosen according to the staircase rules described above. A logistic function was then fitted to the simulated data to determine a simulated threshold estimate. This process was repeated 10000 times (Fig 1). The observed threshold was compared to the distribution of the 10000 simulated thresholds to obtain a standard deviation of threshold estimate. Following Jäkel and Wichmann threshold uncertainty was calculated as the ratio between the simulated standard deviation and the observed threshold (SD/threshold) [4].

In simulation studies the bias is defined as the difference between the simulated threshold and the true model threshold. In our experimental data, the subject’s true threshold is unknown but our best approximation is the estimated threshold after the maximum number of trials. Therefore, bias and precision of empirical thresholds were estimated by comparing the estimated thresholds after a subset of trials with the estimated threshold after the maximum number of trials (80). Bias is defined as the difference between both and precision is defined as the absolute value of the bias.

Last, for each subset of trials the average trial duration and the total experiment duration was calculated.

Fitting of psychometric functions in simulations and real data was done in MATLAB R2014a (Mathworks) using the fminsearch function, and statistical analyses were performed in R [19].

## Results

### Simulations

**Fig 4:**
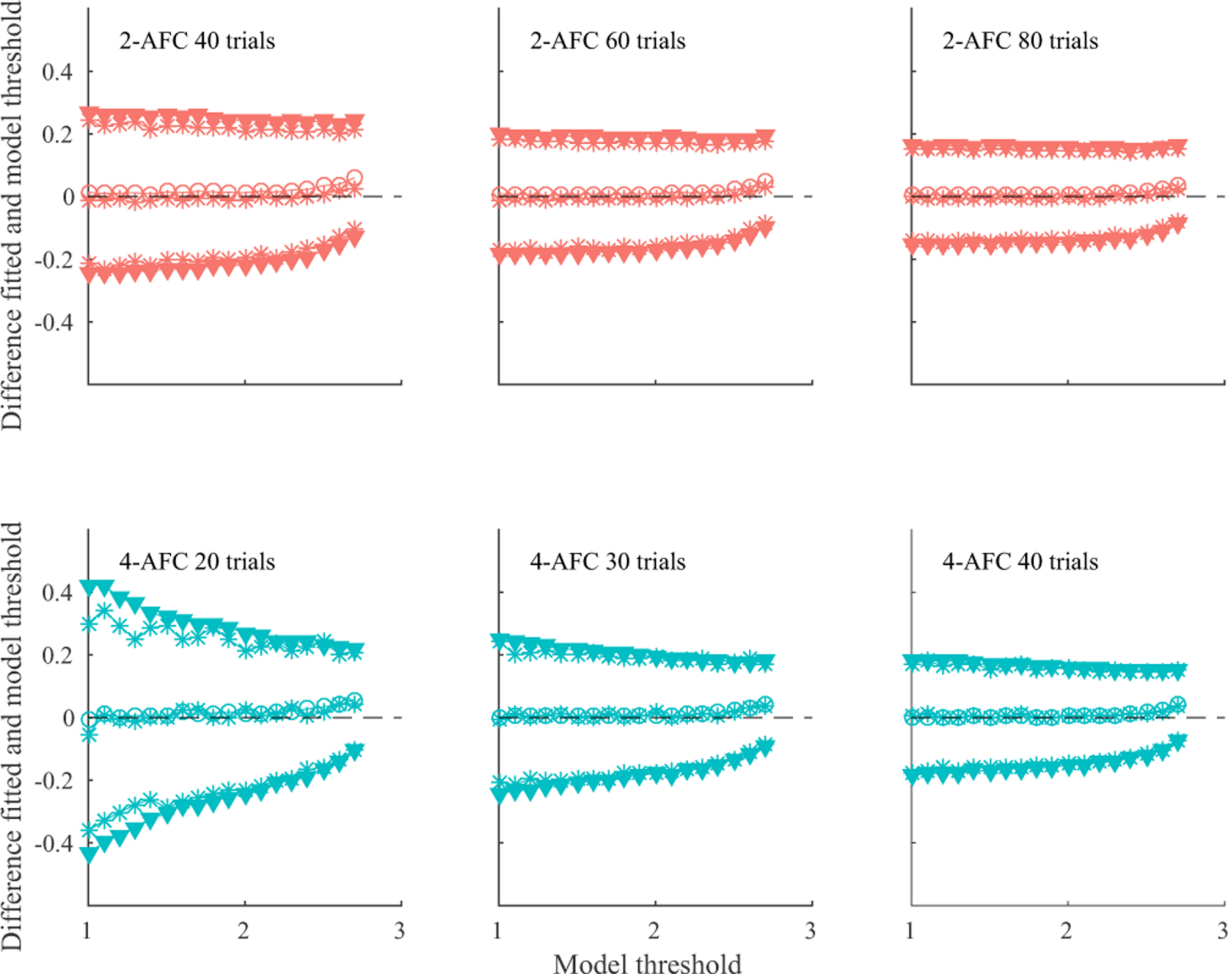
Simulation results for 2-AFC (top) and 4-AFC (bottom). Each panel depicts the difference between the estimated thresholds and the model thresholds as a function of the model threshold (all in log_10_ arcsec). The average difference, plotted with open circles, is centred on zero, indicating no bias. Filled triangle symbols represent one standard deviation below and above the average difference. The asterisks show the median (around zero) and the 16^th^ and 84^th^ percentile. That the median and 16^th^ and 84^th^ percentiles respectively overlap with the mean and one SD below and above the mean indicate that the differences follow a normal distribution. The top row shows results for the 2-AFC task for a selection of number of trials (40, 60, and 80 trials). The bottom row shows results for the 4-AFC task for half the number of trials as the 2-AFC task (20, 30, and 40 trials).

**Fig 5:**
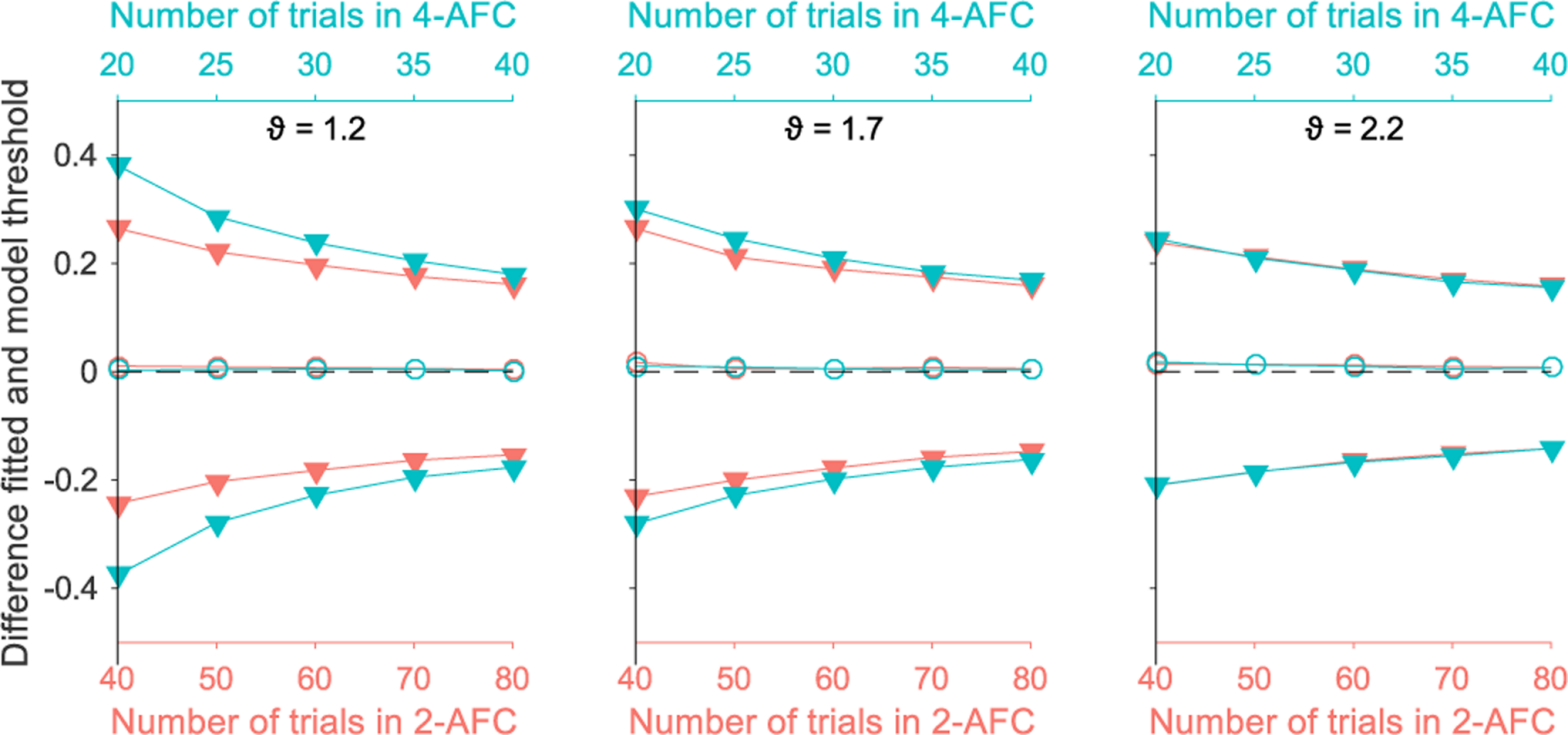
Simulation results. Results for 2-AFC (in red) and 4-AFC (in blue) with half the number of trials as the 2-AFC task are superimposed in the same graph. Each panel depicts the difference between the estimated threshold and the model threshold as a function of number of trials. The model threshold increases from the left to the right panel. The average difference, plotted with open circles, is centred on zero, indicating no bias. Filled triangle symbols represent one standard deviation below and above the average difference.

Fig 4 shows the difference between the estimated threshold by fitting and the true model threshold as a function of the model threshold. Results are plotted for three example trial numbers. Similar results were obtained for the other trial numbers tested. On average, estimated thresholds were very similar to the model thresholds, indicating no bias (open circle symbols in Fig 4). This was true for all tested model thresholds, trial numbers, and tasks. Precision is reflected in the standard deviation of the bias distribution (filled triangle symbols in Fig 4). The standard deviation decreased with an increasing number of trials, indicating better precision (compare panels from left to right in Fig 4). Precision was lower with fewer trials (≤ 20 trials) in combination with low model thresholds (left lower panel in Fig 4). This is a result of the staircase procedure. The staircase starts at a disparity of 3 log_10_ arcsec and comes down in only small steps of 0.15 log_10_ arcsec. This means only few trials around the low model threshold are included in staircases of 20 trials or less. This reduces the precision of the estimate thresholds in these simulations. For any combination of number of trials and model thresholds, precision was always higher in the 4-AFC task than in the 2-AFC task with equal number of trials (compare top left panel with bottom right panel in Fig 4). Similar levels of bias and precision between 2-AFC and 4-AFC tasks are obtained with double the number of trials in the 2-AFC task than in the 4-AFC task (e.g., compare middle top panel with middle bottom panel in Fig 4, also see Fig 5).

In summary, our simulations indicate that overall, bias is close to zero on both tasks independent of number of trials. However, the precision depends strongly both on the number of trials and the true threshold. Precision increases with increasing trial numbers. For all but the lowest thresholds (i.e. good stereoacuity), a 4-AFC task achieves a given precision with half the number of trials as a 2-AFC task. For low thresholds, a 4-AFC task has poorer precision with half the number of trials as a 2-AFC task.

### Empirical data

**Fig 6:**
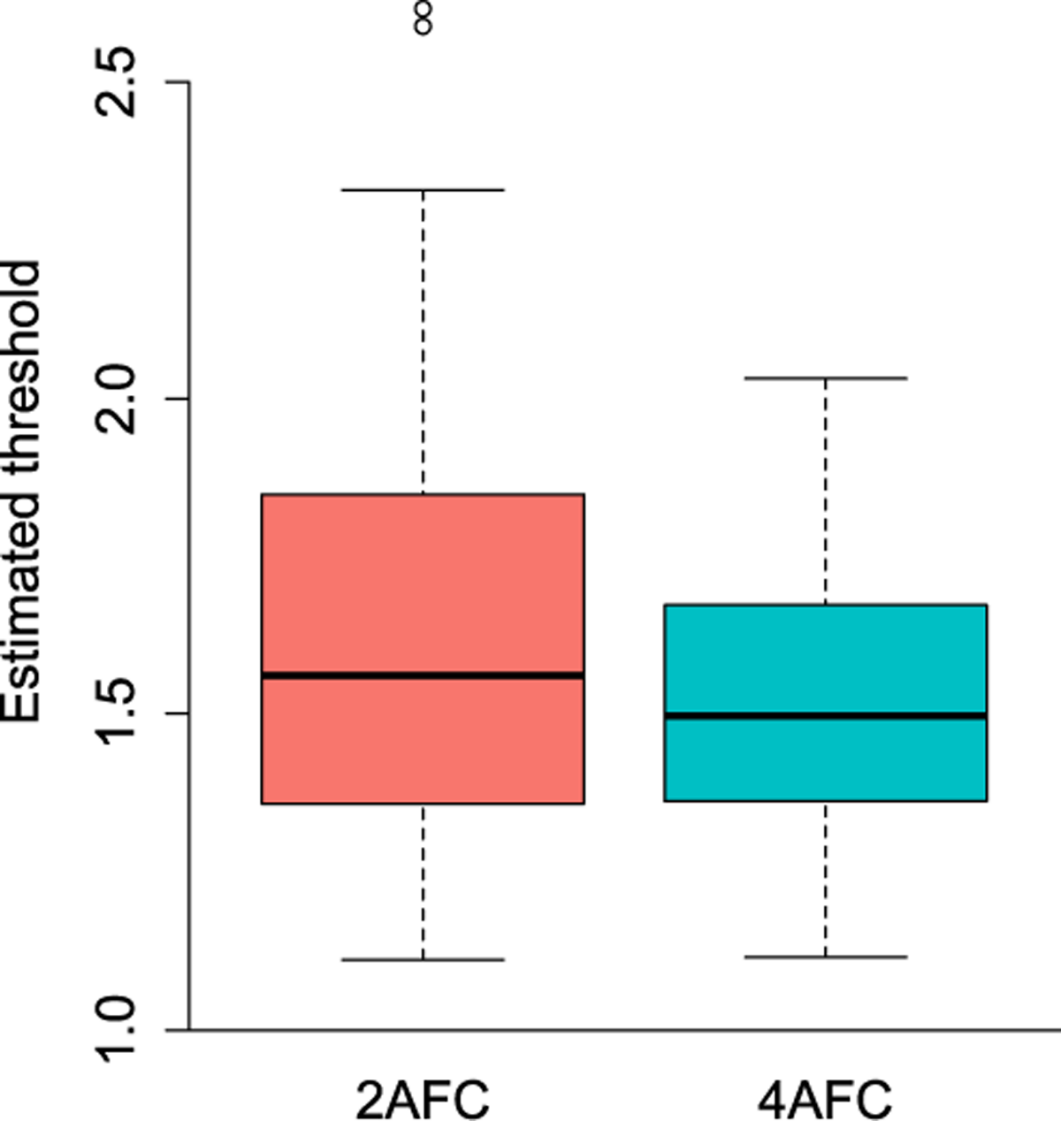
Boxplot of stereothresholds (log_10_ arcsec) in 2-AFC and 4-AFC task (thresholds below 500 arcsec only).

The empirical data are available at figshare with doi: 10.6084/m9.figshare.5939080. For the maximum number of trials (80), multiple linear regression with Task and Age as predictors indicated no significant effect of Task (t(82) = -1.78, *p* = .08) or Age (t(82) = -1.25, *p* = .21) on stereothreshold. It is well documented that stereoacuity depends on age [20–23], but presumably our sample had too small a size and age-range to pick this up. The average stereothreshold was 1.68 log_10_ arcsec in the 2-AFC and 1.69 log_10_ arcsec in the 4-AFC task (Fig 6). The overall regression model explained 3% of the variance in the data (F(2,82) = 2.16, *p* = 0.12, adjusted R^2^ = .03). To meet the assumptions of linear regression, outliers with a stereothreshold at or above 500 arcsec were excluded.

**Fig 7:**
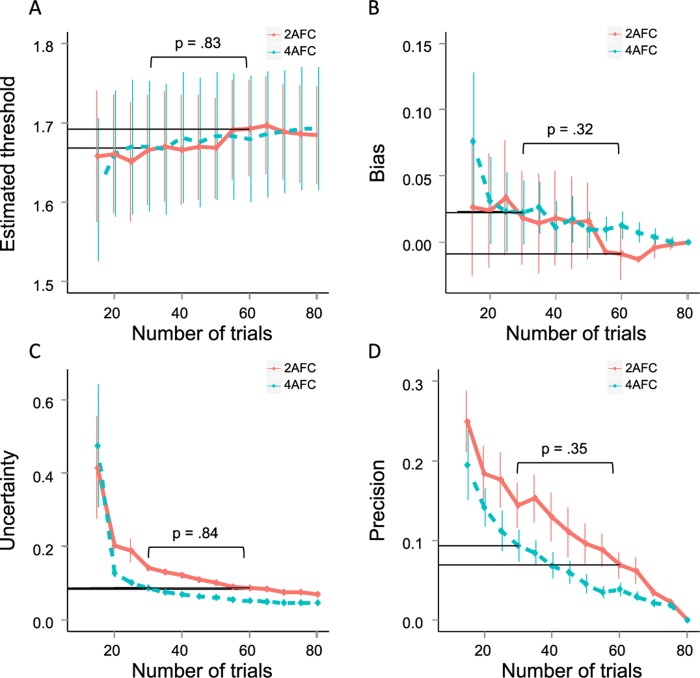
Average threshold estimates, uncertainty, precision and bias for the 2-AFC task (red full line) and the 4-AFC task (blue dotted line). Error bars indicate 1 standard error above and below the average. Threshold uncertainty was calculated as the ratio between the simulated standard deviation and the threshold estimate. Bias and precision of threshold estimates were estimated by comparing the estimated thresholds after a subset of trials with the estimated threshold after the maximum number of trials (80). Bias is defined as the difference between both and precision as the absolute value of the bias.

Fig 7 shows the threshold, bias, uncertainty, and precision for different numbers of trials. Fig 7A shows the mean of the stereothresholds in log units, across participants, as a function of the number of trials completed. Although we are using the same proportion of correct responses (0.75) to obtain the corresponding stereothreshold, we have not found a threshold difference between 2-AFC and 4-AFC tasks. There is also no trend for threshold estimates to increase or decrease with number of trials. This is as expected given that, as we showed in the Simulation study, the average bias should be near zero regardless of the number of trials. Fig 7B shows the estimated bias, based on comparing each subject’s threshold estimate after n trials with their threshold estimate after all 80 trials.

Fig 7C and 7D show the uncertainty of the threshold estimates (defined at the ratio between the standard deviation and the estimated threshold after 80 trials, also known as the relative standard deviation or coefficient of variation, [4] and the precision of the threshold estimate (defined as the absolute value of the difference between the estimated threshold after n trials and after 80 trials). Both decrease with an increasing number of trials, similar to an improvement in precision with increasing number of trials in the Simulation study.

**Fig 8:**
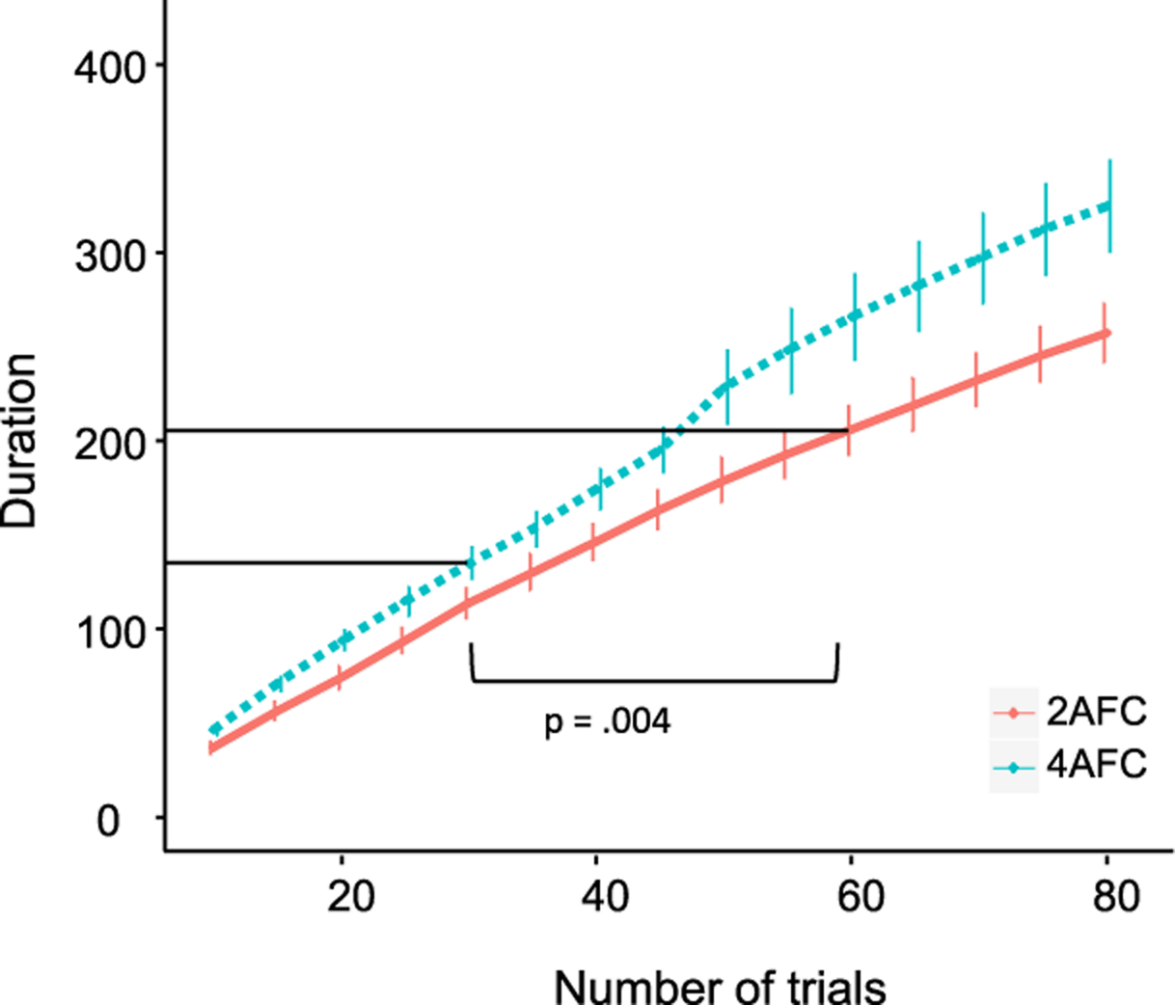
Experiment duration for different trial numbers, for the 2-AFC task (red solid line) and the 4-AFC task (blue dotted line).

Because for each subject we estimated thresholds for the first 15, 20, 25, etc. number of trials, the thresholds are not independent of each other and can therefore not be included in the same statistical analysis. We can however compare thresholds resulting from different number of trials collected with different tasks. In the statistical analyses, we focused on comparing 30 trials in the 4-AFC task with 60 trials in the 2-AFC task. Descriptive statistics for all trial numbers can be found in Figs 7 and 8. Limiting ourselves to these trial numbers reduces the chances of a Type I error. The choice of 30 and 60 trials was informed by our simulation results that indicated that (1) trial numbers below 25 have reduced precision with low threshold estimates, (2) bias and precision are similar with double the number of trials in the 2-AFC than in the 4-AFC task, and (3) we needed to minimize the number of trials in working with children. Because test assumptions were met, no subjects were excluded for the subsequent analyses. We did not observe a significant difference between the estimated threshold after controlling for age (ANCOVA: F(2,89) = 0.6289, *p* = .54, mean 2-AFC = 1.69, mean 4-AFC = 1.67), nor its uncertainty (ANCOVA: F(2,89) = 0.0497, *p* = .95, mean 2-AFC = 0.085, mean 4-AFC = 0.086), bias (ANCOVA: F(2,89) = 0.5434, *p* = .58, mean 2-AFC = -0.009, mean 4-AFC = 0.022), or precision (ANCOVA: F(2,89) = 0.5345, *p* = .59, mean 2-AFC = 0.07, mean 4-AFC = 0.09). This suggests that a 4-AFC task with half the number of trials as a 2-AFC task results in similar thresholds, threshold uncertainty, bias, and precision.

However, we observed a highly significant difference in the duration of the experiment after controlling for age (ANCOVA: F(2,89) = 9.489, *p* < .001, mean 2-AFC = 206.20 sec, mean 4-AFC = 135.37 sec, Fig 8), indicating completing 30 trials in a 4-AFC task takes considerably less time than completing 60 trials in a 2-AFC task even though the average trial duration in the 4-AFC was significantly longer than in the 2-AFC experiment (mean 2-AFC = 3.44 sec, mean 4-AFC = 4.51 sec, ANCOVA: F(2,89) = 4.682, *p* = .01).

## Discussion

In the current study, we showed that for children aged around 10 years completing a small number of trials (<100), estimated stereothresholds, uncertainty, bias, and precision are similar in a 4-AFC task compared to a 2-AFC task with double the number of trials. Individual trials on a 4-AFC task do take around 30% longer than trials on a 2-AFC task, but this increase is not enough to offset the advantage of halving the number of trials required. Accordingly, a 4-AFC task is more time efficient and is therefore recommended.

We did not observe any significant difference in the 75% thresholds measured with the 2- vs 4-AFC tasks after 80 trials. This is perhaps surprising, given that standard signal detection theory would predict a difference [3]. A stronger signal–i.e. larger disparity—would be expected to be required for performance of 75% correct on a 4-AFC where chance is 25%, than on a 2-AFC task, where chance is already 50%. Since in our study the two tasks were performed by different subjects, inter-individual differences may have obscured a difference in thresholds. A d’ value of 0.954 corresponds to 75% correct in a 2-AFC task and 54% correct in a 4-AFC task, meaning that the 75%-threshold in a 2-AFC task should be the same as a 54%-threshold in a 4-AFC task if the model by Green and Swets is correct [3]. In fact, Jäkel and Wichmann [4] actually observed lower instead of higher 62.5% thresholds in a 4-AFC contrast detection task than 75% thresholds on a 2-AFC task, indicating that the theoretical predictions may not be correct. The lack of a difference in thresholds between the 2-AFC and 4-AFC tasks does not affect our conclusions, since our study was designed to make within-task, within-subject comparisons of thresholds, bias, uncertainty, and precision.

Our study adds to the research on efficiency of alternative forced-choice paradigms in psychophysical research. Our results with children are in accordance with simulation studies comparing a 4-AFC task with a 2-AFC task. Leek, Hanna, and Marchal [8] found better accuracy, less variability and highest efficiency with 4-AFC task. Bi, Lee, and O’Mahony [9] showed 4-AFC is more powerful than 2-AFC. Like Jäkel and Wichmann [4] we found a 4-AFC task to be most efficient in naive observers. With respect to efficiency, both studies point in the same direction, in favour of a 4-AFC task.

In the domain of stereoacuity, Schmidt, Maguire, Moore, and Cyert [24] showed a higher testability in 3 to 3.6 year olds with the 2-AFC Stereo Smile Test than with the 4-AFC Preschool Randot. These children are far younger than those tested in the current study, raising the possibility that 2-AFC may have particular advantages in preschool children. Additionally, the nature of the both tasks is rather different: in the Stereo Smile Test, children detect a smiley face, while in the Preschool Randot children need to identify shapes. So, the higher testability of the Stereo Smile could easily be due to factors other than the lower number of alternatives.

Our findings have implications for clinical practice too. They suggest that 4-AFC paradigms like used in Frisby (Frisby Stereotests, Fulwood, United Kingdom) or TNO (Lameris, Ede, Netherlands) stereotests are more efficient than a 2-AFC task like Frisby Screener (Frisby Stereotests, Fulwood, United Kingdom). We are currently adapting our experimental 4-AFC task to a new clinical stereotest in the form of a game that is presented on a parallax-barrier autostereoscopic tablet that monitors viewing distance. Our task has the additional advantage of using an efficient adaptive staircase to achieve an accurate threshold with the minimum number of trials. For details, please visit https://research.ncl.ac.uk/asteroid/ [25].

## Conclusions

In this study, we compared a 2-AFC and a 4-AFC task for measuring stereoacuity in children with a limited number of trials. Simulations were run and experimental data were collected. We observed that a 4-AFC task with half the number of trials as a 2-AFC task results in the same estimated stereothresholds, uncertainty, precision, and bias, while takes less time to complete. We conclude that 4-AFC is recommended over 2-AFC when measuring stereoacuity in children.

## References

[1] Kingdom FAA, Prins N. Psychophysics A Practical Introduction. London: Elsevier; 2010.

[2] Morgan MJ, Melmoth D, Solomon JA. Linking hypotheses underlying Class A and Class B methods. Vis Neurosci. 2013;30: 197–206. 10.1017/S095252381300045X 24476966 PMC4131156

[3] Green DM, Swets JA. Signal Detection Theory and Psychophysics. New York, NY: Wiley; 1966.

[4] Jäkel F, Wichmann FA. Spatial four-alternative forced-choice method is the preferred psychophysical method for naïve observers. J Vis. 2006;6: 1307–22. 10.1167/6.11.13 17209737

[5] García-Pérez MA, Alcalá-Quintana R. Sampling Plans for Fitting the Psychometric Function. Span J Psychol. 2005;8: 256–289. 10.1017/S113874160000514X 16255393

[6] Hou F, Lesmes L, Bex P, Dorr M, Lu Z-L. Using 10AFC to further improve the efficiency of the quick CSF method. J Vis. 2015;15: 2 10.1167/15.9.2 26161631 PMC4581618

[7] Shelton BR, Scarrow I. Two-alternative versus three-alternative procedures for threshold estimation. Percept Psychophys. 1984;35: 385–392. 10.3758/BF03206343 6739274

[8] Leek MR, Hanna TE, Marshall L. Estimation of psychometric functions from adaptive tracking procedures. Percept Psychophys. 1992;51: 247–256. 10.3758/BF03212251 1561050

[9] Bi J, Lee HS, O’Mahony M. d’ and variance of d’ for four-alternative forced choice (4-AFC). J Sens Stud. 2010;25: 740–750. 10.1111/j.1745-459X.2010.00301.x

[10] Betts J, McKay J, Maruff P, Anderson V. The development of sustained attention in children: the effect of age and task load. Child Neuropsychol. 2006;12: 205–221. 10.1080/09297040500488522 16837396

[11] Serrano-Pedraza I, Herbert W, Villa-Laso L, Widdall M, Vancleef K, Read JCA. The Stereoscopic Anisotropy Develops During Childhood. Investig Opthalmology Vis Sci. 2016;57: 960 10.1167/iovs.15-17766 26962692 PMC4788095

[12] García-Pérez MA. Forced-choice staircases with fixed step sizes: asymptotic and small-sample properties. Vision Res. Pergamon; 1998;38: 1861–1881. 10.1016/S0042-6989(97)00340-4 9797963

[13] Serrano-Pedraza I, Vancleef K, Herbert W, Woodhouse M, Read JCA. Determination of the slope of the psychometric function for different stereoacuity tasks. J Vis. The Association for Research in Vision and Ophthalmology; 2016;16: 838 10.1167/16.12.838

[14] Wichmann F a, Hill NJ. The psychometric function: I. Fitting, sampling, and goodness of fit. Percept Psychophys. 2001;63: 1293–313. Available: http://www.ncbi.nlm.nih.gov/pubmed/11800458 11800458 10.3758/bf03194544

[15] Brainard DH. The Psychophysics Toolbox. Spat Vis. 1997;10: 433–6. 9176952

[16] Pelli DG. The VideoToolbox software for visual psychophysics: transforming numbers into movies. Spat Vis. 1997;10: 437–42. 9176953

[17] Kleiner M, Brainard DH, Pelli DG. What’s new in Psychtoobox-3? Perception. 2007;36: 14.

[18] Serrano-Pedraza I, Vancleef K, Read JCA. Avoiding monocular artifacts in clinical stereotests presented on column-interleaved digital stereoscopic displays. J Vis. Association for Research in Vision and Ophthalmology; 2016;16: 13 10.1167/16.14.13 27846341 PMC5114011

[19] R Development Core Team. R: A language and environment for statistical computing. Vienna, Austria: R Foundation for Statistical computing; 2011.

[20] Fox R, Patterson R, Francis EL. Stereoocuity in Young Children. Invest Ophthalmol Vis Sci. 1986;27: 598–600. 3957579

[21] Oduntan AO, Al-Ghamdi M, Al-Dosari H. Randot stereoacuity norms in a population of Saudi Arabian children. Clin Exp Optom. 1998;81: 193–197. 12482318 10.1111/j.1444-0938.1998.tb06734.x

[22] Garnham L, Sloper JJ. Effect of age on adult stereoacuity as measured by different types of stereotest. Br J Ophthalmol. 2006;90: 91–5. 10.1136/bjo.2005.077719 16361675 PMC1856927

[23] Schmid M, Largo RH. Visual acuity and stereopsis between the ages of 5 and 10 years—A cross-sectional study. Eur J Pediatr. 1986;145: 475–479. 10.1007/BF02429046 3493140

[24] Schmidt PP, Maguire MG, Moore B, Cyert L, Vision in Preschoolers Study Group. Testability of preschoolers on stereotests used to screen vision disorders. Optom Vis Sci. 2003;80: 753–7. 14627942 10.1097/00006324-200311000-00012

[25] Read JCA, Vancleef K, Serrano-Pedraza I, Morgan G, Sharp C, Clarke M. ASTEROID: Accurate STEReoacuity measurement in the eye clinic. Perception. 2015;44: 75–76.

